# Ecological and historical factors behind the spatial structure of the historical field patterns in the Czech Republic

**DOI:** 10.1038/s41598-022-12612-8

**Published:** 2022-05-23

**Authors:** Václav Fanta, Jaromír Beneš, Jan Zouhar, Volha Rakava, Ivana Šitnerová, Kristina Janečková Molnárová, Ladislav Šmejda, Petr Sklenicka

**Affiliations:** 1 Faculty of Environmental Sciences, Czech University of Life Sciences Prague, Kamýcká 129, 165 00 Prague – Suchdol, Czech Republic; 2 Institute of Archaeology, Faculty of Philosophy, University of South Bohemia, Branišovská 31, 370 05 České Budějovice, Czech Republic; 3 Laboratory of Archaeobotany and Palaeoecology, Faculty of Science, University of South Bohemia, Na Zlaté stoce 3, 370 05 České Budějovice, Czech Republic; 4 Prague University of Economics and Business, W. Churchill Sq. 1938/4, 130 67 Prague 3 – Žižkov, Czech Republic; 5 Department of Anthropology, Faculty of Arts, University of West Bohemia, Univerzitní 8, 306 14 Plzeň, Czech Republic

**Keywords:** Environmental social sciences, Anthropology, Archaeology

## Abstract

Historical field systems are an essential part of the traditional cultural landscape of societies with primarily agricultural subsistence. They embody many functions and values, as they affect the productional, ecological and hydrological functioning of the landscape, its cultural values, the way people perceive the landscape, and their impact on present-day farming. As an aspect of the historical landscape, field systems are a topic investigated in landscape archaeology, environmental studies, historical geography, landscape ecology, and related disciplines. Historical field systems can form many complex spatial structures, shapes and patterns. This paper focuses on identifying environmental and historical/cultural driving forces during the formation and the historical development of various field pattern types. We worked with 523 settlements established in the medieval to the early modern period (approx. 900–1600 AD) in the present-day Czech Republic. We have determined the proportions of different field pattern types in the examined cadastres and have statistically compared them with a variety of environmental and geographical predictors. Our results indicate a strong influence of environmental predictors (terrain undulation, cadastre size), the impact of specific historical events and associated social changes (e.g. land confiscations by the state in the seventeenth century), and a significant relationship between field pattern types and settlement layout types. Furthermore, we have observed the different adaptations of field pattern types to similar environmental conditions, as well as the impact of social and political factors on the processes of landscape formation. Our paper provides the first detailed analysis of the geographical distribution of traditional field systems on the scale of an entire modern state, and emphasizes the importance of transdisciplinary research on cultural landscapes.

## Introduction

### General introduction

Historical field systems and their remnants are an essential part of the historical cultural landscape. As a typical representation of the “combined work of nature and of man”^1^, their pattern constitutes the image of the organically evolved cultural landscape and forms an integral part of a country’s cultural heritage^2–5^. The history of field systems also significantly impacts the ecological function of present-day farming landscapes. Elements of these systems, especially historical field margins, control the physical, chemical and biological fluxes in the landscape^6^, mitigate soil erosion^7^, stabilize the landscape’s hydrological regime^8,9^, reduce pesticide drift and fertilizer misplacement^10,11^, and serve as a buffer against nitrates and for water protection^12^. Furthermore, the remnants of historical field systems contribute to biodiversity conservation, serving as a habitat and a conduit for wildlife^2,7,13,14^. From the standpoint of landscape ecology, the remnants of traditional field systems form specific landscape features^15^, which incorporate the spatial structure of the landscape, historical works of man, archaeological complexity and various ecological functions^6,16,17^. We also want to stress that the ecological functions of historical field systems and hedgerows have dramatically changed since the end of traditional agriculture^18^. Last but not least, the historical field systems as key macrostructures also form the image of the landscape and its aesthetical values^7,19^.

Historical field systems, and knowledge gained from studying them, also play a significant role in modern sustainable agriculture and landscape planning^20,21^. For example, with the ever-increasing impact of climate change, the hydrological function of remnants of historical field systems and their ability to mitigate erosion and retain water have becomes critical (cf.^22,23^). In addition, the historical field pattern reflects the land ownership structure^15^ and social relationships between members of historical society^24^, which makes it an essential topic for historians, archaeologists and historical geographers^25–30^. In terms of spatial archaeology, historical field systems form one of the largest landscape macrostructures (or landscape “superartifacts” sensu Chang^31^), retaining information about their chronology, function, and environmental characteristics^32–34^. The historical field pattern also co-creates a country’s cultural identity^35,36^. Unfortunately, these landscape structures have tended to disappear or to have been abandoned from the middle of the twentieth century onwards^37,38^.

To sum up, historical field systems and their remnants contribute with many values to the present-day cultural landscape: (a) they take their part in forming the “image of the landscape”, (b) they affect its ecological and hydrological functions, (c) they are historical witnesses of ancient times, and they reflect agriculture in the past and land-ownership traditions, and (d) they have a strong influence on present-day farming. In brief, the remnants of historical field systems can be remarkable from many points of view. This paper will focus on various types of spatial arrangement of historical field patterns in the Czech Republic, their driving forces and their spatial distribution.

### Primary forms of historical field systems

Transformation of the landscape into organized field systems has taken place in many parts of the world^39,40^. The most significant form of historical field systems are well visible *terraced fields*. This phenomenon is probably related to the origin of historical state organization, and it prevailed in agrarian usage of foothills and mountainous areas with more prominent dynamics of relief^41^. Terracing is an ancient technology; the oldest known (proto)terraces (*paddy fields*) were already constructed in the Neolithic period in China^42^. The oldest evidence of terraces is recorded from the Levant around 6000 BC^43,44^. At a Neolithic site in Croatia, excavation of terraces associated with the site has been reported^45^. The non-agricultural terraces were also used in the famous Neolithic site of Lepenski Vir in Serbia^46^. Some studies admit the existence of terraces in the Aegean area in the Bronze Age^47^. Further evidence of terraces is known from South-East Asia around 3000 BC^46^. The oldest examples outside the Mediterranean area and South-East Asia have been reported from the Andes around 2400 BC^48^. In the Alpine Region and the Maya Lowlands, the oldest terracing practices date back to the Iron Age^49^. All these examples correlate well with the introduction of agriculture.

In addition to their agricultural function, terraced fields help retain water and prevent soil erosion^15,49,50^. Terraced fields are found on all continents. They are present in Asia^51^, South America^52^, Central and North America^53^, and the mountainous regions of Africa^54^ and the Near East^32^. In Europe, terraced fields are located mainly in the Mediterranean area^33,55^, in the Alpine region^56^, and in Central Europe^15,53^. There are several types of terraces, but all are built with two elements—the terraced platform and the terraced slope. A traditional terraced slope is a dry stone wall structure, or a slope reinforced with stones grubbed out during farmland cultivation and later covered with earth and grassed over^57^. Modern terraces are usually grassed slopes. Terraces are typically used as an agricultural space, frequently for growing grapes and other fruit^57–59^, olives^60^, vineyards^58^ and for grazing^58^. These terraces are typical, for example, in France^61^, Slovenia^57^, Portugal^62^, Spain^63^ and Greece^64^.

The second significant form of the historical field system is characterized by visible boundaries of the parcels^39^. These field systems are called *bocage* or *hedgerow patterns* in western Europe^6^, *Flur* in Germany^65–68^, and *plužina* in the Czech Republic^2^. The boundaries between parcels were mostly marked by small walls, banks, steep slopes, pathways, or by lines of trees or bushes. These landscape elements also provided important secondary functions, e.g. they affected the surface water flow or served as a source of firewood^15^. In the present-day landscape, these traditional agricultural field patterns mostly appear as mosaics of small-scale arable fields, pastures, orchards or vineyards. Typically, the fields in these landscapes are defined by hedgerows composed of trees and shrubs, often unmanaged or with a low level of management (cf.^13,69^). In some parts, there can be small woodland patches for obtaining timber. This landscape structure results from the historical development of agriculture, and it constitutes a traditional agroforestry system, which is typical for Central Europe^15,70,71^.

The third significant form of the historical field system is so-called *open fields*, well-known from Great Britain^17,30,72^. In these extensive systems, individual parcels are not separated by walls or other boundaries^15^. Renes^73^ explains that the long and narrow shapes of these fields were generated using a heavy plough, which allowed only limited manipulation. This agrarian landscape setting following older prehistoric field systems^74^ probably appeared in the tenth century and lasted until the sixteenth century^15,73^.

### Historical field pattern typologies

Several typological schemes have differentiated these field systems according to their spatial division, shape, size, terrain, etc. The individual national/regional typologies reflect the types present in the respective country. Authors from the Mediterranean region usually describe types of terraced fields. In contrast, Central European authors focus on field systems with visible boundaries (e.g. *Flur* in Germany, *plužina* in the Czech Republic). In some cases, these typologies may overlap.

There are two ways to differentiate agricultural terraces: horizontal layout (planform) and vertical profile. The horizontal layout can strictly follow the contour lines, can form braided structures or lynchets^75^, or, alternatively, they can compose wave-like terraces^49^ or off-contour terraces^76^. Moreover, Brown et al.^75^ identified regular box-like terrace structures and, together with Wei et al.^49^, referenced pocket/half-moon shaped terraces. Pocket terraces also have a wall-free “spontaneous” version^76^. Based on the vertical profile, authors have identified four^77^, six^75^ or seven types^49^ of terraces. The divisions are primarily based on the construction material^77^ or on the terrain gradient of the levelled part of the terraces^49,75,77^. The levelled part can also have a reversed slope or can include a ditch to increase the water retention capacity^78^.

**Figure 1:**
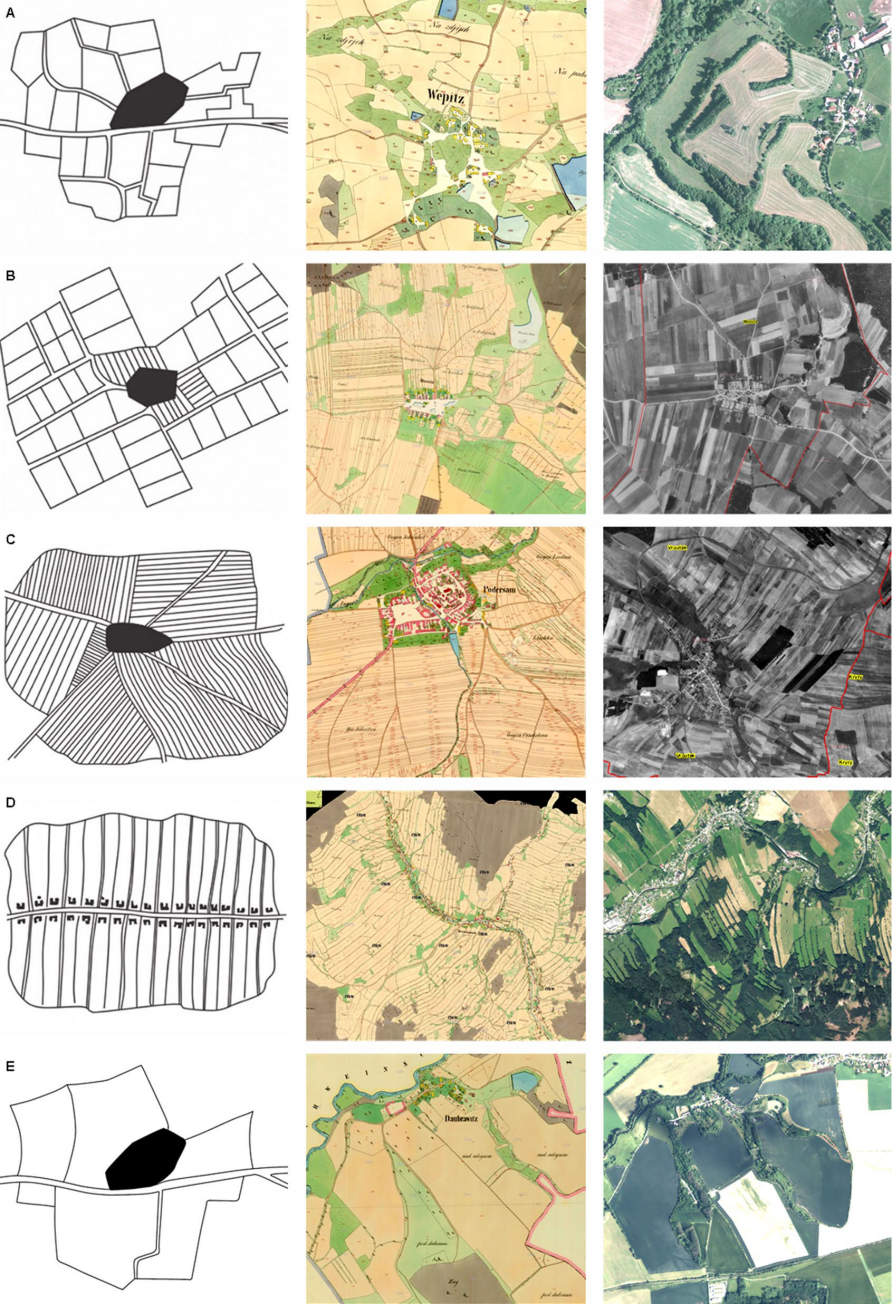
Five types of historical field pattern. (**A**) Segmental plužina (irregular, blocky structure; Czech: *úseková*), (**B**) plužina of consolidated/unconsolidated segments (regular, narrow, short strips arranged in many rectangular quadrangles; Czech: *scelené/dělené úseky*), (**C**) sectional plužina (narrow and medium-length strips parallel to each other, arranged into large irregular blocks; Czech: *traťová*), (**D**) croft plužina (very long parallel strips following each homestead; Czech: *záhumenicová/délková*), (**E**) without internal division, and others. First column: schematic drawing of the field pattern [drawings (**A**–**D**) by Šitnerová et al.^15^, after Černý^81^, drawing E by Václav Fanta, on the base of Černý’s^81^], second column: *Imperial Imprints of the Stable Cadastre* mid-nineteenth century maps^82^, third column: historic (**B**,**C**) or recent (**A**,**D**,**E**) aerial photographs^82^.

Unlike terraced fields, field systems with visible boundaries exhibit great diversity in their shapes, as they are constructed in less steep terrain. These field systems can be divided into many types, according to the spatial division of land ownership (individual homesteads or noble estates) and the terrain where they were built^79^. There are several typologies of these plužina field systems: Löw and Míchal^79^ identified six types of plužina field pattern, Láznička^80^ identified seven types, while the broadest classification was introduced by Černý^81^, who divided the plužina field system into nine types. These typologies differ in details and in terminology. However, all of them recognize the following basic types: (a) an irregular, blocky structure, often called segmental or blocky plužina (Fig. 1A), (b) narrow and medium-length strips parallel to each other, arranged into large irregular blocks, called sectional plužina (Fig. 1C), and (c) very long parallel strips adjacent to each homestead, called croft or longitudinal plužina (Fig. 1D). Other types of field patterns with visible boundaries have also been reported from the Mediterranean landscape, e.g. more or less regular flat fields and sloping fields, which occur mostly in plain terrain with fertile soils^77^. Other authors from the same region make a distinction between regular *vs* irregular non-terrace field patterns^76^. A different typology based on social classification of historical field patterns has been recently proposed by Klír^24^. His extraordinary approach is focused on the social and economic relations of the traditional village society and within the members of this society.

Experts suggest that the diversity of historical field pattern types reflects various factors affecting their genesis, historical development and environmental conditions^17,39,66^. Several authors have emphasized the influence of age of origin. Žemlička^83^ claims that there are differences between the field pattern type in ancient settlement area (i.e. regions with settlement continuity since the Neolithic period^84^) and in areas colonized in the medieval period. According to Vermouzek^85^, Klápště^86^, Čulíková^87^ and Šitnerová et al.^15^, the irregular, blocky field pattern (segmental plužina; Fig. 1A) is bound to the early medieval period, while narrow and medium-length strips of fields (sectional plužina; Fig. 1C) are characteristic of the high medieval period. Sadravetzová^88^ found segmental plužina predominantly in the high medieval period, and croft plužina (Fig. 1D) in the late medieval and modern period. However, her research was based on dating from written records, which systematically provides dating that is younger than archaeological dating^89,90^. She also surveyed a sub-mountainous borderland area, which was probably colonized later than the inland of the country. Other authors agree about the influence of age of origin, but without detailed specification (e.g.^83^). Although the cited references are either small-scale studies^85,87,88^ or review papers/books without a detailed statistical investigation^15,83,86^, we can conclude that segmental plužina is probably the oldest type, that sectional plužina is younger, and that croft plužina is the youngest type. Examples from Scandinavia show that the time when a regular form of field pattern was introduced varied between regions^91^. The transition from segmental plužina to sectional plužina was perhaps caused by the introduction of new agricultural tools, mainly the heavy plough, which required longer parcels^15,86,92–94^.

Various authors have recognized the existence of a direct relationship between the village layout and the field system type^15,24,95,96^, but only Sadravetzová^88^ suggests direct links between them (a solitary house or a scattered hamlet—scattered plužina, village green settlements—segmental plužina, a village with hides/*Waldhufendorf* settlement—croft plužina). Many papers and books have presented the expected influence of environmental conditions, mainly terrain properties and soil quality^15,83,85,97,98^ (for further references see^99^), however without providing proper statistical evidence. Lucke et al.^100^ report that specific geological conditions (and thus soil properties) affected distinctive field patterns in the Steigerwald region in Germany. Similarly, the occurrence of terraced landscapes in Slovenia has been strongly influenced by geological conditions (mainly flysch and carbonate rocks), by altitude (predominantly 100–600 m), and by southern aspect^101^; the shape of the Slovenian terraces and their vertical section is also affected by the terrain inclination^102^. Unfortunately, these studies did not focus on field pattern types. A clear connection between field pattern type and altitude was found by Sadravetzová^88^ in her local study (segmental and sectional plužina at a lower altitude, croft plužina at higher altitudes). In France, four typical regional forms of terraces have been identified^61^.

Based on his earlier micro-regional study^103^, Žemlička^83^ claims that the number of landlords who simultaneously owned parts of a village in the thirteenth and fourteenth century still affected the field type recorded in the nineteenth century cadastral maps. According to his findings, villages owned by a single landlord in the medieval period mainly still had sectional plužina in the 19th-century maps. In contrast, villages owned by two or more landlords in medieval times had segmental plužina in the nineteenth century. He explains this phenomenon as follows: For the redesign of the field pattern of a village owned by more than one landlord, it was necessary to make a deal among these landlords—which was obviously too complicated. Such villages thus retained their older segmental plužina.

Unfortunately, some of the studies mentioned here are small-scale (local or regional), or lack proper statistical evidence, or do not deal with detailed field pattern typology. However, they suggest possible relationships that can be statistically tested.

The historical field patterns have also been interpreted as a reflection of social structures of the adjacent village society^24,104,105^. Besides the already discussed environmental factors, Born^106^ stresses the influence of agrarian technologies, demography, social and economic factors^24^. Other authors underline the role of local institutions in shaping historical field patterns^24,107^.

### Spatial distribution of historical field patterns

The spatial extent of historical field patterns has been studied in many European countries: in the Czech Republic^24,35,108^, France^61^, Italy^109^, Slovakia^71,110^, Slovenia^57,101,111^ and historical regions of Saxony (today part of Germany), Silesia (today part of the Czech Republic and Poland), Galicia/Halychyna (today part of Poland and Ukraine)^65^. These studies are on different levels of detail: some surveyed the whole area of a country^111^, while others identified only a number of areas with remnants of historical field patterns^35^. Some authors also tried to determine the spatial distribution of various types of historical field patterns^24,35,65^, but none succeeded on the level of an entire country.

### The focus of our study

In this paper, we decided to focus on the situation in the Czech Republic. This region is characterized by the availability of high-quality historical maps depicting field patterns^82,112,113^, and also by a long tradition of archaeological research on medieval settlements^86,114–118^, on landscape and environmental archaeology^25,28,89,119,120^ and on historical field systems^8,15,28,34,121–126^. The Czech Republic is also characterized by high geological diversity^127^, by diversity of landscape types^128,129^ and by diverse vernacular architecture^130^. Such diversity may have contributed to the historical development of field pattern types.

Within the last millennium, the cultural landscape of Central Europe has undergone several dramatic transformations that have changed the settlement structure: medieval colonization and field pattern redesign^86,131,132^, the 15th-century religious wars, which destroyed many villages^133–135^, the Thirty Years’ War, which resulted in a decline in population by one third, economic losses, land abandonment and property confiscations^23,136–140^, eighteenth to nineteenth-century rational redesign of the field ownership structure^27,85,141^, an industrial revolution with the rapid expansion of cities^142^, mid-twentieth century forced collectivization of agriculture, extensive changes in land use, the destruction of traditional field patterns, and ruining of private land ownership by the communist regime^37,143–148^, as well as land consolidation activities, some of which changed the small-scale historical field pattern into large blocks of arable land^149^. At the present time, some parts of the agricultural landscape are being abandoned for various reasons^38^. Each of these changes has affected the image of the landscape and its functioning.

The plužina field patterns, which prevailed in the Czech cultural landscape at the time of its medieval colonization and redesign, have gradually changed. Some plužinas disappeared along with villages that were lost in wars and gradually became re-forested^125,150^. Others, especially those in the most fertile areas, were transformed into larger arable fields, which can be seen on Stable cadastre maps dating back to the first half of the nineteenth century. However, in the 1950s, plužina field patterns were still predominant in large parts of the Czech territory^79^. This situation changed fundamentally in the second half of the twentieth century, when many of these landscapes disappeared due to agricultural intensification or due to agricultural extensification and gradual re-afforestation^2^.

The rapid loss of plužinas, along with their outstanding historical, natural and aesthetic values, has led to efforts to gain a deeper understanding of these valuable landscape segments, and to conserve them. However, there still remains a lot that is unknown about plužinas. As we have noted above, the historical field patterns have different shapes and structuring. Although the role of environmental or cultural/historical factors in determining the type of plužina prevailing in a specific location has been discussed for decades, precise identification of the “driving forces” supported by statistical evidence is still missing, as is the spatial distribution of individual field pattern types on a nationwide scale. This paper will therefore focus on identifying ecological, geographical and historical factors that could have shaped the spatial structure of the historical field patterns and the spatial distribution of individual types of field patterns within the Czech Republic. In other words, why does the cultural landscape of Central Europe have its present appearance? What formative factors can be statistically linked to its development?

## Methods

### Data collection

Since it is expected that the field pattern types differed across the centuries^83,87,88^, we wanted to include the age of the villages as one of the predictors. Because the dating of the origin of historical settlements by widely-available written sources is highly biased^89,90^, we decided to work with archaeologically dated settlements only. We used a database from our previous research^89^, which contains a subset from the *Archaeological Database of Bohemia*^151^. This subset includes 527 medieval and early modern age settlements dated by precise archaeological methods (for a detailed description of the data filtering, please refer to the Methods section in the respective paper). In four cases, it was impossible to determine the field pattern types of the respective settlements (either there were no fields in the cadastre, or the old cadastral maps were missing). Such cases were excluded from the dataset. As a result, we worked with 523 settlement sites (for their geographic distribution, see Fig. S1). Unfortunately, archaeological data in sufficient quality and data structure are available only for the western part of the country (the historical region of Bohemia), and not for the entire country. This research therefore covers approximately two-thirds of the Czech Republic only.

The types of field patterns were derived from the *Indication sketches of the Stable Cadastre* (German: *Urmappe des Franziszeischen Katasters*^152^, Czech: *indikační skici stabilního katastru*), a set of old cadastral maps from the first half of the nineteenth century^82^. These maps are the oldest historical source available for the whole country depicting the field pattern structure. The scale of these maps is 1 : 2 880, and they are characterized by high geodetical accuracy. These maps, as a highly respected source, have been routinely used in many historical, geographical and ecological studies^2,28,153–155^.

Within recent decades, experts have identified several typological systems of historical field patterns^79–81,156,157^. Most typological systems contain six to nine types of field pattern. We took the most distinctive types from Černý’s^81^ typology, and we grouped other very similar types. As a result, we obtained four basic types (A: segmental, B: consolidated/unconsolidated segments, C: sectional, D: croft plužina), and we also added one more type (E) for “others/unspecifiable/without internal division” (Fig. 1).

In most cases, the cadastre of a single village contains two or more types of field patterns. In each cadastre, we therefore described all types of field patterns that were present, and then we estimated their relative extent, rounded to tens of percentage points (example: in the village of *Mochov*, the following types of field pattern were identified: type A 50%, type B 30%, type E 20%). Of course, the sum of the relative extents of all types in one cadastre is 100%. We also recorded the *field pattern diversity* as the number of field pattern types within one cadastre.

All predictors (independent variables) were derived for points representing the centre of the respective village or town. We worked with four environmental variables: *Altitude* data was taken from the SRTM digital terrain model^158^. *Terrain undulation* was measured as the average degree of slope within a 4 km radius from the village/town, while the slope was derived from the SRTM model^158^. A description of the *geological* bedrock was derived from the geological map^159^ and was simplified into four categories (metamorphic / igneous / sedimentary /quaternary). We thank geologist Radana “Radouch” Kavková for her assistance with this simplification. The specific natural *soil fertility* values (0 = worst, 100 = best) were calculated from the soil units data^160^, using the methodology described in Bečvářová et al.^161^.

Next, we operated with historical/geographical variables: The *archaeological dating* was obtained from the Archaeological Database of Bohemia^151^. The *ancient settlement area* (area inhabited continuously since the Neolithic) was defined as cadasters with Neolithic findings obtained from the Archaeological Database of Bohemia^151^. We thank archaeologist Jiří “Bumča” Bumerl for his assistance with the archaeological data filtering. To observe the possible impact of important medieval monasteries, we measured the distance to *monasteries* founded in the twelfth and thirteenth centuries^162,163^. The *Cadastre area* was calculated from the current geographical data^164^. *Settlement type* data was derived from the database of historical settlements by Kuča^165^. This predictor describes the layout composition of towns and villages in 8 different types (villages with regular hides, orthogonal village green locations, non-orthogonal village green locations, small villages, modern parcel villages and dispersed villages, other villages, modern settlements, historical towns). We grouped the “orthogonal village green locations” and the “non-orthogonal village green locations” into a single group called “village green locations”; we also grouped “small villages”, “modern settlements”, and “other villages” into a single group called “other villages” (for a detailed description of this typology, see^126^). *Confiscates* were derived from Semotanová and Cajthaml^166^ as areas with a forced change of landlord in the seventeenth century. [In the first half of the seventeenth century, the emperor from the House of Habsburg confiscated the properties of revolting members of the nobility (primarily protestants) and installed foreign (catholic) nobility in vacant dominions. This process involved a substantial part of the country].

We also added a variable describing historical subsistence strategies: *Agriculture* represents the agricultural regions in the sixteenth century^167^, dividing the country into three areas: wheat areas (commercial agriculture production), flax areas (linen production, secondary non-agrarian production), and other areas.

Last but not least, we would like to stress that this list of variables does not include all possible predictors. We think it would be fascinating to have, for example, data on the medieval ownership structure, and to test Žemlička’s^83^ hypothesis on the influence of the number of landlords. Unfortunately, it is impossible to gather such data for the whole country.

All dependent and independent variables are arranged in Table 1. The geographical data were processed in QGIS 3.22.0 software (https://qgis.org/en/site/) and SAGA 7.8.2 software (https://saga-gis.sourceforge.io/en/index.html)^168^.

**Table 1:** All variables used in the study.

Variable name	Description	Data source
**Dependent variables (response)**		
*Diversity*	Number of field pattern types within one cadastre	Land Survey Office^82^
*Type A*	The relative extent of the respective field pattern type in the cadastre (0–100 %)	
*Type B*		
*Type C*		
*Type D*		
*Type E*		
**Environmental independent variables (predictors)**		
*Altitude*	Meters above sea level	GISAT^158^
*Terrain undulation*	Average degree of slope within a 4 km radius	
*Geology*	Geological bedrock, simplified	Czech Geological Survey^159^
*Soil fertility*	Relative soil fertility (0 = worst, 100 = best)	State Land Office^160^, Bečvářová et al.^161^
**Historical/geographical independent variables (predictors)**		
*Archaeological dating*	Age of the settlement	Institute of Archaeology of the Czech Academy of Sciences Prague^151^
*Ancient settlement area*	Presence or absence of Neolithic findings in the cadastre	Institute of Archaeology of the Czech Academy of Sciences Prague^151^
*Monasteries*	Distance to medieval monasteries	Purš^162^, Hrnčiarová et al.^163^
*Confiscates*	Areas confiscated in the seventeenth century	Semotanová and Cajthaml^166^
*Settlement type*	Type of layout composition	Kuča^165^
*Cadastre area*	Square meters	Arcdata Praha^164^
**Historical subsistence strategies**		
*Agriculture*	agricultural/subsistence strategies in sixteenth century	Klír^167^

### Statistical analysis

We assess the effect of our predictors on the dependent variables using regression analyses. The choice of the regression model depends crucially on the nature of the dependent variable. It might feel natural to treat *diversity* (the number of different types of field pattern) as a count outcome; however, its values are restricted to the 1–5 range, due to our classification of field patterns. We therefore adopt two alternative modelling strategies: (i) truncated Poisson regression, with truncation points set at 0 and 6, (ii) an ordinal logistic regression that considers *diversity* a general 1–5 ordinal scale.

In the regressions that explained the relative extent of field patterns A–E, the outcome is a *composition*, or a *multinomial fractional response*. The presence of zeros (in most cadastres, not all patterns are represented) rules out the use of the standard Dirichlet regression approach. We therefore opted for the fractional multinomial logistic quasi-maximum likelihood approach, inspired by Papke and Wooldridge’s^169^ estimator for a (single) fractional response and implemented by Buis^170^.

Our explanatory variables exhibited a mild degree of collinearity; most notable correlations (0.55–0.62) occurred between environmental predictors (*altitude*, *terrain undulation*, *soil fertility*). These correlations are not strong enough to preclude simultaneous use of the variables in a multivariate regression setting. However, in the fractional multinomial logistic regressions, where four coefficients per predictor are being estimated, the effect of collinearity might be more pronounced; therefore, for robustness, we present the results from both bivariate and multivariate regressions. *Geology* was excluded from the multivariate regressions due to a nearly 20% rate of missingness; for the other variables, values were missing in less than 2% of the sample (see Table 2 for sample sizes in bivariate regressions). Several numeric predictors (*terrain undulation*, *altitude*, *cadastre size*, *monasteries*) showed a substantial positive skew; we log-transformed their values prior to all statistical analyses.

**Table 2:** Summary statistics for bivariate and multivariate regressions.

Dependent variable	Proportions of field patterns			Field pattern diversity			
Model	Fractional multinomial logit			Ordinal logit		Truncated Poisson	
	*N*	*p*-value	RVI	*p*-value	RVI	*p*-value	RVI
Terrain undulation (log)	523	< 0.0001	0.998	0.509	0.284	0.025	0.280
Altitude (log)	523	0.0310	0.282	0.116	0.609	0.016	0.423
Soil fertility	516	0.0110	0.014	0.594	0.337	0.023	0.303
Cadastre area (log)	523	< 0.0001	0.588	< 0.001	0.997	< 0.001	0.950
Archaeological dating	523	< 0.0001	0.050	0.046	0.589	0.010	0.393
Monasteries (log)	523	0.1352	0.034	0.708	0.290	0.026	0.274
Confiscates	515	0.0013	0.212	0.638	0.267	0.024	0.284
Settlement type	514	< 0.0001	1.000	0.041	0.064	0.019	0.054
Geology	405	< 0.0001		0.661		0.146	

*N* the number of observations in bivariate regressions; *p*-value overall model *p*-value in bivariate regressions; *RVI* relative variable importance, i.e., sum of the Akaike weights across all models containing the given covariate in the all-subset regressions (*geology* was not included in the all-subset regressions).

Finally, we used the *relative variable importance* (RVI) measure to rank predictors in terms of the weight of evidence for their inclusion in the best model. A predictor’s RVI, also referred to as the parameter weight, the parameter inclusion probability or the proportion of evidence^171–173^, is obtained by (i) running the regressions with all possible subsets of the available predictors, (ii) calculating the Akaike weight for each estimated model, and (iii) summing the Akaike weights across all models that included the predictor of interest. RVI is bounded between 0 and 1; thresholds of 0.4^171^ or 0.5^174^ have been recommended to separate important variables and unimportant variables.

All regression analyses were conducted in Stata 17.0 (https://www.stata.com/). The user-contributed commands *fmlogit*^170^, *miinc*^173^, and *estout*^175^ were used for the fractional multinomial logistic regression, for the RVI calculation, and for the regression tables, respectively. The geographical interpolation for Fig. 4 was performed in SAGA software^176^ using the *universal kriging* tool.

## Results

According to Table 2, the most significant variables affecting the proportions of field pattern types were *settlement type* (RVI = 1.000), *terrain undulation* (RVI = 0.998), and *cadastre area* (RVI = 0.588). These variables affected almost all field pattern types. The influence of other variables had less impact on the overall variability. Some variables did not affect any field pattern type at all: distance to medieval *monasteries* (see Table 2) and *ancient settlement area* (*p*-value from the bivariate regression is 0.092). The field pattern diversity was influenced by one variable only: the *cadastre area* (RVI = 0.950).

**Figure 2:**
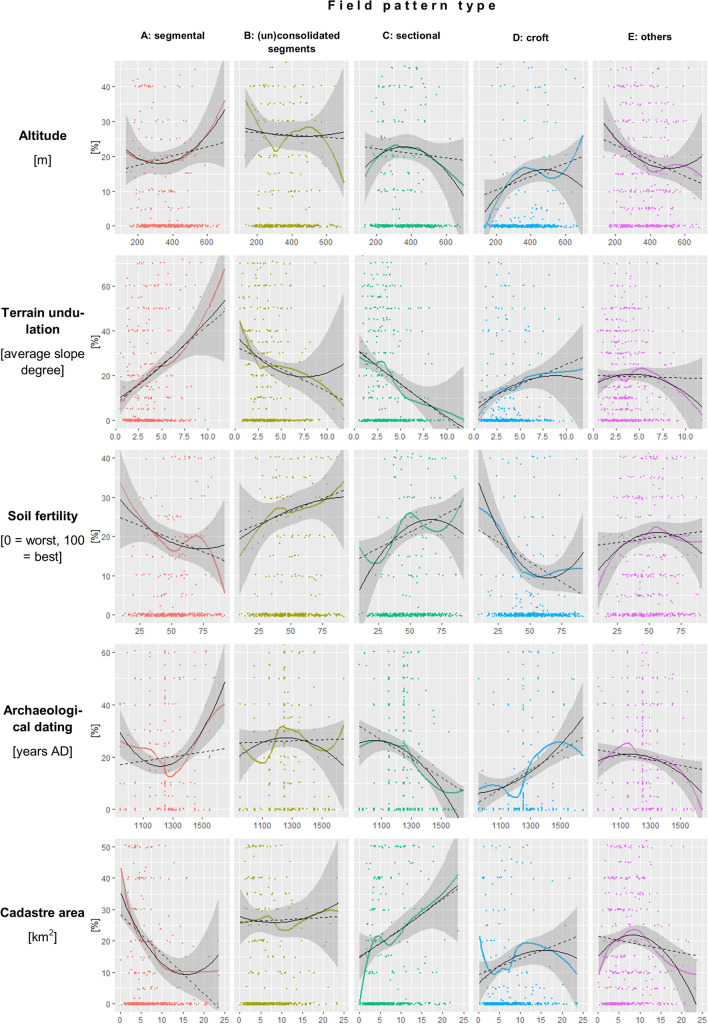
Influence of continuous variables. The percentages refer to the proportion of the specific field pattern type in the examined cadastres. The descriptions of variables and their units of the x-axes are on the left side of the respective panels.

**Figure 3:**
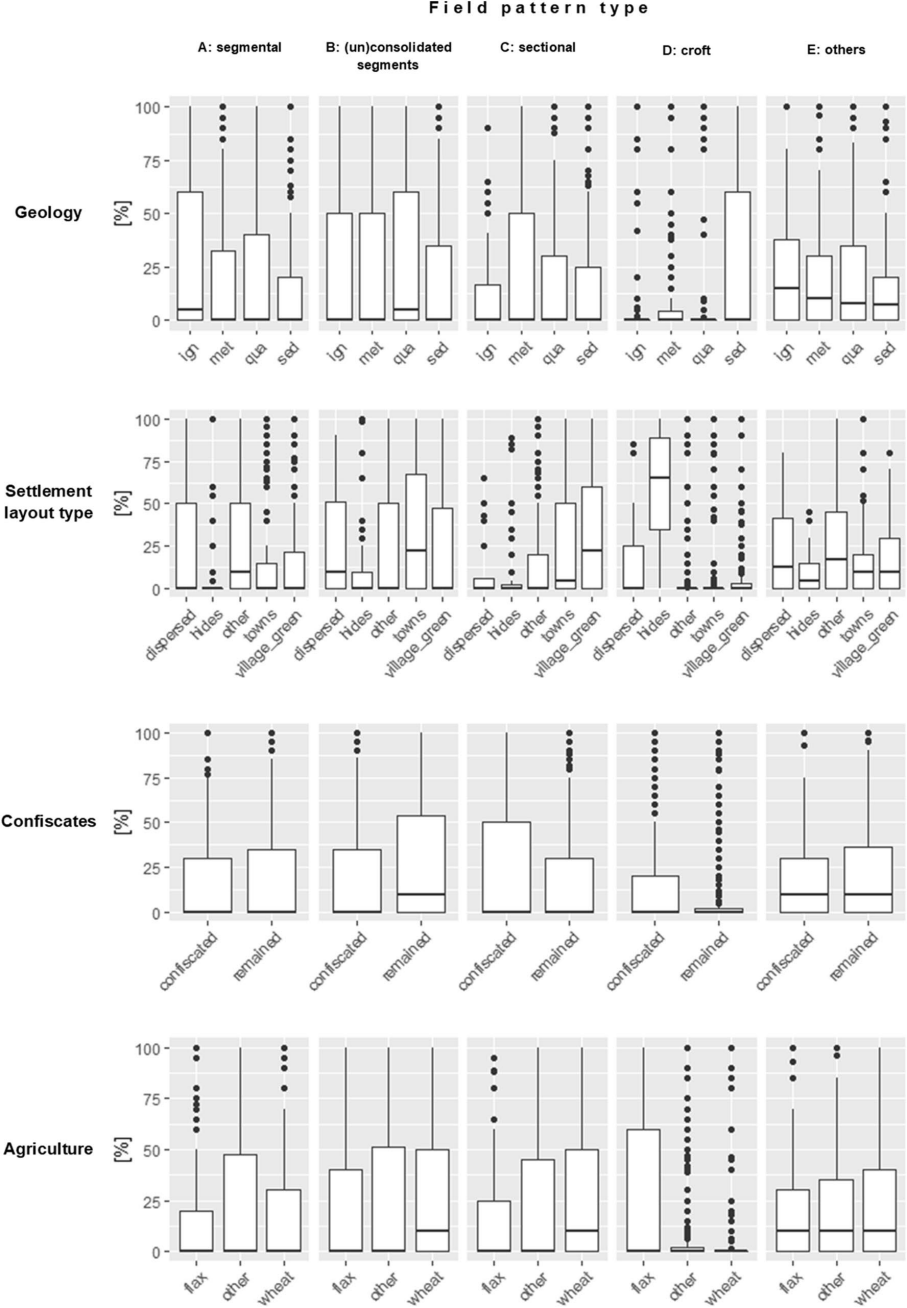
Influence of categorical variables. The percentages refer to the proportion of the specific field pattern type in the examined cadastres. The descriptions of variables of the x-axes are on the left side of the respective panels.

Field pattern *type A* (segmental plužina) was associated with very rugged *terrain* (*p* < 0.001), small *cadastre area* (*p* < 0.001), preferably igneous but definitely non-sedimentary *geological* bedrock (see Fig. 3) and specific types of *settlement layout type* (“village green locations” and “other”). The influence of *altitude* was insignificant; however, Fig. 2 suggests a possible preference for higher locations. Both Table 3 and Fig. 2 agree with the preference for poor *soil fertility* of this type, but the *p*-value is above 0.05. According to Fig. 2, this type was connected either with very old (pre-1200 AD) or with very young (post-1500 AD) *archaeological dating*. It was not affected by *confiscates*.

**Table 3:** Average marginal effects from bivariate and multivariate regressions: fractional multinomial logit explaining the proportions of field patterns.

Regression	A: segmental		B: (un)cons. segments		C: sectional		D: croft		E: others	
	AME	SE	AME	SE	AME	SE	AME	SE	AME	SE
**Terrain undulation (log)**										
Bivariate	11.51***	(0.0239)	− 8.253**	(0.0260)	− 9.605***	(0.0212)	6.503**	(0.0214)	− 0.158	(0.0196)
Multivariate	14.36***	(0.0305)	− 10.33**	(0.0353)	− 11.39***	(0.0306)	2.922	(0.0235)	4.445	(0.0253)
**Altitude (log)**										
Bivariate	4.320	(0.0383)	− 2.210	(0.0424)	− 1.873	(0.0347)	7.029*	(0.0289)	− 7.266*	(0.0326)
Multivariate	− 9.482	(0.0487)	9.951	(0.0591)	13.55**	(0.0521)	− 3.768	(0.0388)	− 10.25*	(0.0419)
**Soil fertility**										
Bivariate	− 13.29	(0.0790)	13.00	(0.0892)	16.44*	(0.0724)	− 20.64*	(0.0802)	4.490	(0.0585)
Multivariate	− 10.42	(0.110)	0.845	(0.125)	11.86	(0.106)	0.0803	(0.0887)	− 2.368	(0.0796)
**Cadastre area (log)**										
Bivariate	− 7.339***	(0.0185)	− 1.146	(0.0197)	4.896**	(0.0165)	3.625	(0.0192)	− 0.0358	(0.0120)
Multivariate	− 6.139***	(0.0182)	− 3.239	(0.0214)	6.209***	(0.0188)	0.814	(0.0207)	2.354	(0.0157)
**Archaeological dating**										
Bivariate	1.287	(0.0114)	− 0.0455	(0.0106)	− 3.123***	(0.00857)	2.980***	(0.00824)	− 1.097	(0.00725)
Multivariate	0.656	(0.0120)	0.817	(0.0122)	− 1.507	(0.0101)	1.027	(0.00803)	− 0.993	(0.00759)
**Monasteries (log)**										
Bivariate	− 1.612	(0.0154)	− 0.491	(0.0176)	− 2.628	(0.0143)	3.677	(0.0201)	1.054	(0.0143)
Multivariate	− 1.413	(0.0154)	− 0.0109	(0.0203)	− 1.734	(0.0163)	1.945	(0.0146)	1.212	(0.0136)
**Confiscates**										
Bivariate	− 3.531	(0.0269)	− 6.280*	(0.0310)	5.533*	(0.0264)	7.376**	(0.0239)	− 3.097	(0.0204)
Multivariate	− 1.215	(0.0265)	− 6.314*	(0.0317)	5.939*	(0.0258)	3.827	(0.0201)	− 2.237	(0.0206)
**Settlement type**										
*Village with hides*	Ref.		Ref.		Ref.		Ref.		Ref.	
*Village green*										
Bivariate	9.002*	(0.0434)	15.45**	(0.0555)	21.01***	(0.0509)	− 53.19***	(0.0542)	7.720**	(0.0285)
Multivariate	10.39*	(0.0460)	10.87	(0.0591)	18.06***	(0.0525)	− 46.77***	(0.0571)	7.453*	(0.0316)
*Dispersed*										
Bivariate	15.08	(0.0791)	14.72	(0.0781)	1.032	(0.0554)	− 43.12***	(0.0782)	12.28*	(0.0571)
Multivariate	10.99	(0.0747)	10.34	(0.0775)	4.493	(0.0658)	− 39.68***	(0.0760)	13.86*	(0.0629)
*Towns*										
Bivariate	5.730	(0.0386)	22.54***	(0.0485)	14.44***	(0.0430)	− 49.43***	(0.0539)	6.722**	(0.0241)
Multivariate	7.993*	(0.0398)	23.89***	(0.0553)	8.672*	(0.0417)	− 44.95***	(0.0547)	4.392	(0.0271)
*Others*										
Bivariate	15.45***	(0.0353)	13.26**	(0.0406)	9.238*	(0.0364)	− 52.71***	(0.0499)	14.76***	(0.0238)
Multivariate	13.14***	(0.0347)	10.51*	(0.0470)	10.39**	(0.0394)	− 47.04***	(0.0550)	12.99***	(0.0275)
**Geology**										
*ign*	Ref.		Ref.		Ref.		Ref.		Ref.	
*met*										
Bivariate	− 9.048	(0.0536)	1.019	(0.0525)	10.74**	(0.0414)	1.238	(0.0307)	− 3.945	(0.0384)
*qua*										
Bivariate	− 8.944	(0.0601)	3.976	(0.0625)	4.873	(0.0453)	2.946	(0.0423)	− 2.851	(0.0467)
*sed*										
Bivariate	− 14.63**	(0.0510)	− 2.842	(0.0510)	5.688	(0.0387)	20.52***	(0.0399)	− 8.739*	(0.0365)

(i) To enhance readability, *archaeological date* and *soil fertility* were divided by 100 prior to running the regressions. (ii) the standard errors, in parentheses, were obtained from regression estimates via the delta method. (iii) *p < 0.05, **p < 0.01, ***p < 0.001.

Field pattern *type B* (plužina of consolidated/unconsolidated segments) was associated with flat *terrain*, and with specific types of *settlement layout type* (“village green locations”, “towns” and “other”), and with dominions that remained in possession of the original landlords after the Thirty Years’ War (variable *confiscates*). The *soil fertility* might play a role (a slight preference for more fertile soils). This type was not affected by *altitude* (the proportion of type B within the cadasters seems to be independent of the altitude), *geology*, *archaeological dating* (the percentage of type B did not change over the centuries) or *cadastre area* (clearly insignificant).

Field pattern *type C* (sectional plužina) was associated with flat *terrain* (*p* < 0.001), big *cadastre area*, dominions *confiscated* in the seventeenth century, older (mostly pre 1300 AD) *archaeological dating*, good *soil fertility*, specific types of *settlement layout type* (“village green locations”, “towns” and “other”), and preferably metamorphic *geological* bedrock. The influence of *altitude* is unclear: in Table 3, the bivariate model shows insignificant negative dependence, while the multivariate model shows significant positive dependence, and Fig. 2 suggests relatively mild negative dependence.

Field pattern *type D* (croft plužina) was primarily associated with one specific type of *settlement layout type* (“villages with hides”) and with sedimentary *geological* bedrock. The other variables are problematic, since the bivariate and multivariate models offer different results (see Table 2). Nevertheless, based on Fig. 2, we can say that there was probably a preference for higher *altitude* (the graph suggests that areas over 300–500 m have a higher percentage of type D than lower areas), more *undulating terrain*, poor *soil fertility* (according to the bivariate model, this was apparently quite an important variable), very young (late) *archaeological dating* (post-1300–1400 AD), and maybe the dominions *confiscated* in the seventeenth century. This type was not affected by the *cadastre size*.

Field pattern *type E* (without internal division, and others) was associated with lower *altitude*, igneous *geological* bedrock and specific types of *settlement* layout (all except “villages with hides”). This field pattern was not affected by *terrain undulation*, *soil fertility*, *archaeological dating*, *confiscates* or *cadastre size*.

According to Table 4, the *diversity* was positively affected by the *cadastre area* (a more extensive cadastre area led to higher field pattern diversity). Fig. S2 suggest a possible but insignificant negative influence of *archaeological dating* and *altitude* (older sites and lower altitude should lead to greater diversity). The other variables had no significant effect.

**Table 4:** Coefficient estimates for bivariate and multivariate regressions explaining field pattern diversity.

Regression	Ordinal logit		Truncated Poisson	
	Coeff.	SE	Coeff.	SE
**Terrain undulation (log)**				
Bivariate	− 0.0963	(0.146)	− 0.0254	(0.0688)
Multivariate	0.0657	(0.739)	0.0256	(0.780)
**Altitude (log)**				
Bivariate	− 0.364	(0.232)	− 0.103	(0.109)
Multivariate	− 0.438	(0.182)	− 0.152	(0.322)
**Soil fertility**				
Bivariate	0.252	(0.472)	0.0347	(0.219)
Multivariate	0.101	(0.882)	− 0.0139	(0.964)
**Cadastre area (log)**				
Bivariate	0.450***	(0.110)	0.162**	(0.0507)
Multivariate	0.401**	(0.002)	0.134*	(0.026)
**Archaeological dating**				
Bivariate	− 0.116*	(0.0580)	− 0.0345	(0.0267)
Multivariate	− 0.101	(0.134)	− 0.0259	(0.399)
**Monasteries (log)**				
Bivariate	0.0373	(0.0996)	0.00564	(0.0467)
Multivariate	0.0611	(0.566)	0.0184	(0.714)
**Confiscates**				
Bivariate	0.0781	(0.166)	0.0571	(0.0766)
Multivariate	0.0281	(0.871)	0.0333	(0.678)
**Settlement type**				
*Village with hides*	Ref.		Ref.	
*Village green*				
Bivariate	0.164	(0.333)	0.0983	(0.160)
Multivariate	0.138	(0.694)	0.103	(0.536)
*Dispersed*				
Bivariate	0.146	(0.461)	0.0738	(0.234)
Multivariate	0.398	(0.409)	0.154	(0.525)
*Towns*				
Bivariate	0.745*	(0.309)	0.303*	(0.148)
Multivariate	0.441	(0.172)	0.202	(0.189)
*Others*				
Bivariate	0.138	(0.278)	0.0822	(0.138)
Multivariate	0.178	(0.548)	0.112	(0.437)
**Geology**				
*ign*	Ref.		Ref.	
*met*				
Bivariate	0.350	(0.285)	0.0964	(0.132)
*qua*				
Bivariate	0.166	(0.330)	0.0291	(0.153)
*sed*				
Bivariate	0.211	(0.281)	0.0401	(0.130)

(i) To enhance readability, *archaeological date* and *soil fertility* were divided by 100 prior to running the regressions. (ii) *p < 0.05, **p < 0.01, ***p < 0.001.

## Discussion

**Figure 4:**
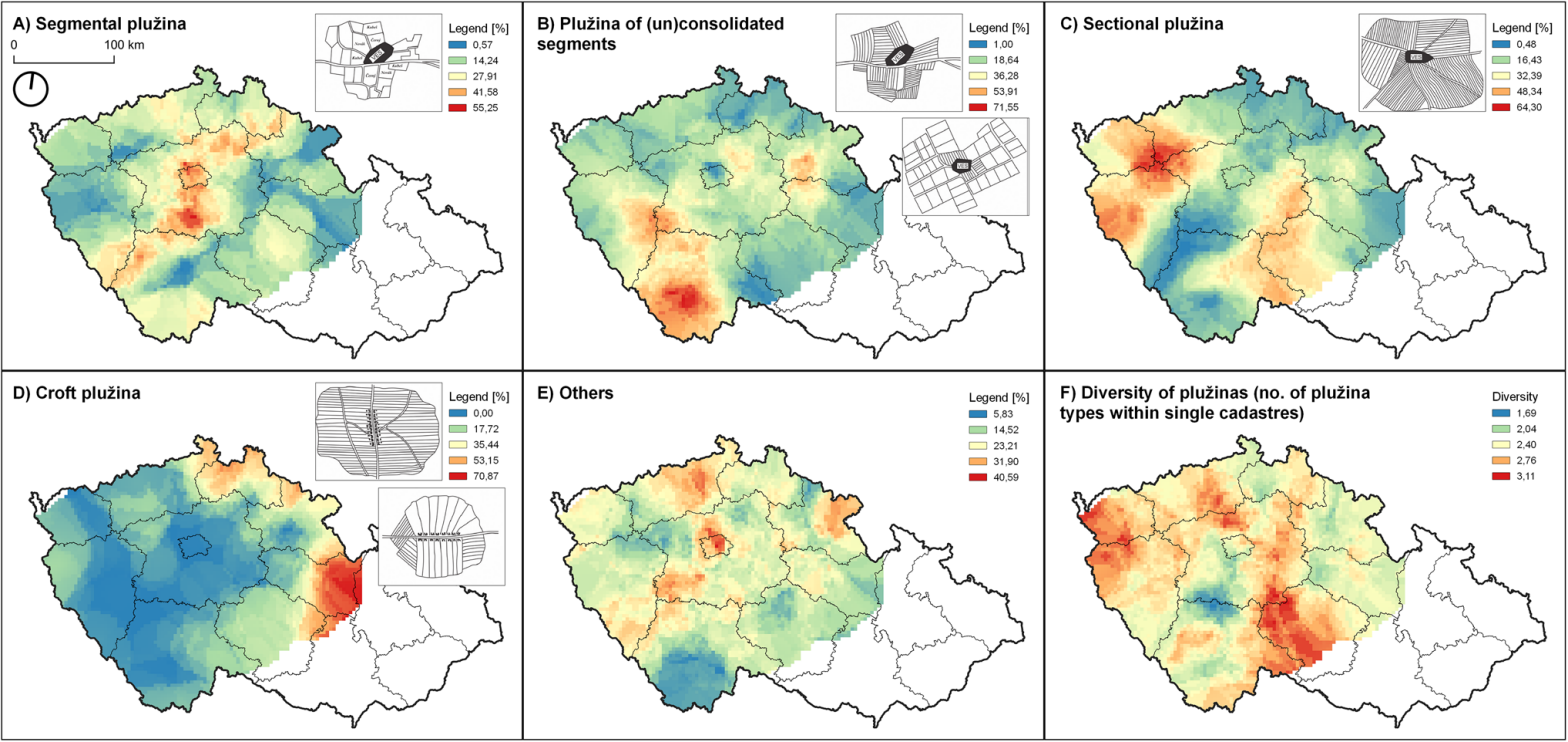
(**A**–**E**) Geographical distribution of field pattern types within Bohemia (western part of the Czech Republic). The percentages refer to the proportion of each field pattern type in the examined cadastres. The field pattern types are shown in the schematic black-and-white drawings. (**F**) Diversity of field pattern types, i.e. the number of field pattern types within examined cadastres. All panels: The original point-based data were interpolated using the *universal kriging* interpolation tool in SAGA software^176^. The eastern part of the Czech Republic (Moravia, Silesia) is not represented because of missing archaeological data. The black-and-white schematic drawings of plužina types in panels (**A**–**D**) are from Šitnerová et al.^15^, after Černý^81^. The map was created by authors using QGIS software (https://qgis.org/en/site/, version 3.22.0) and SAGA software (https://saga-gis.sourceforge.io/en/index.html, version 7.8.2).

The results show that the number of field pattern types within a single cadastre (variable *diversity*) was significantly affected by one predictor only—the size of the cadastre, i.e. the bigger the cadastre area, the greater the diversity. The explanation for this phenomenon is trivial: given the approximately constant count of field pattern types per unit area, a bigger cadastre area offers a higher probability of the occurrence of a specific field pattern type—cf. the “species-area relationship” in ecology^177^, which is however driven by other processes. Surprisingly, no other predictors had a significant effect. Nevertheless, we have identified a remarkable spatial distribution of field pattern diversity across the country (Fig. 4F). Some regions are characterized by relatively high diversity (marked by red colour), while others are curiously homogenous (blue). We hypothesize that this distribution may be affected by other factors not included in our study. We can summarize that the average number of field pattern types within a cadastre varies between two and three. During the pre-industrial period, the cadastre size also directly affected the number of inhabitants in a village by providing the area for agricultural activities, i.e. for their subsistence^137^.

Field pattern *type A* (segmental plužina) was characterized by extreme environmental conditions (higher altitude, extremely rugged terrain, igneous bedrock and low soil fertility). Type A is also associated with tiny cadastres and specific types of settlement layouts (“other”)—however, the latter association is insignificant. Field pattern *type A* is situated at settlements founded either in the early medieval period (pre-1200 AD) or in the modern period (post-1500 AD). It is present primarily in a belt across the whole country (Fig. 4A), which is (in most cases) characterized by harsh conditions. We can summarize that type A (segmental plužina) is present in locations with extreme conditions—environmental and cultural (cadastre size). It is a kind of “extremophile organism”—it can “survive” in situations where other strategies wither away^178–180^. We hypothesize that this field pattern type could initially also have been present in areas with better conditions, but it may have been replaced by type C (sectional plužina), because of type C’s better suitability for medieval agriculture^132,181^. Type A then remained only in areas with bad conditions for agriculture. Type A was frequent at settlements founded in the early medieval period, which corresponds well with previous findings^15,85–88^. The second peak of this type lies in the early modern period. We think that this reflects the late medieval to early modern colonization of highlands and sub-mountainous regions, where other types of field patterns could not be established because of the harsh environmental conditions and limited spatial possibilities. Surprisingly, no terraces (as we know them from the Mediterranean area) have been built (except for the vineyards in Moravia) in the areas with significant terrain undulation (up to 10°). Perhaps it was easier to organize segmental plužina structure than to build terrace walls.

Field pattern *type B* (plužina of consolidated/unconsolidated segments) was characterized by relatively high soil fertility, flat terrain and specific types of settlement layout (mostly “towns”). This type occurs at almost all altitudes and in almost all ages within the study period. The geology and the cadastre area were also insignificant. It is present primarily in southern Bohemia (Fig. 4B). To conclude, this field pattern type prefers relatively flat terrain and higher soil fertility, i.e. good conditions for agriculture. Other variables have limited influence. The shape of this field pattern testifies to rational land consolidation in restricted blocks without significant spatial requirements or splitting the larger fields, initially owned by the nobility, into small fields during the modern period^27,182,183^. As field pattern *type B* is not bound to a specific historical period, it was probably a general method of secondary land consolidation in areas with good agricultural conditions^182,184^.

Field pattern *type C* (sectional plužina) was characterized by optimal conditions for agriculture (lower and medium altitude, flat terrain, very high soil fertility, metamorphic bedrock, and big cadastre area). It is associated with settlements established in the early and high medieval period (pre-1300 AD, almost no occurrence after 1400 AD) and with specific types of settlement layouts (“village green locations”). It is present primarily in western Bohemia (Fig. 4C). This field pattern has much better environmental conditions for agricultural production than type B. It also requires much more space—usually the whole area of the cadastre. Considering the relatively early dating (pre-1300 AD), we suggest two possible ways in which this field pattern was set up: either it was set up in the early medieval period (together with the foundation of the village—see^15,86,87^), or, like type B, it may have been an instrument of secondary land consolidation^132,182,185,186^, motivated by excellent conditions for agriculture.

Field pattern *type D* (croft plužina) was characterized by relatively poor conditions for agriculture (higher altitude, more undulating terrain, sedimentary bedrock, and poor soil fertility). This type is found in settlements established primarily in the late medieval and modern periods (post-1300–1400 AD), corresponding with Sadravetzová^88^. Type D is present almost exclusively under these particular conditions. It is present mainly in the mountains of northern and eastern Bohemia (Fig. 4D). This type is also strictly bound to “villages with regular hides” and to areas with secondary non-agrarian production (flax areas, Fig. 3). The sedimentary bedrock suggests that it occurred primarily in valleys. We can conclude that this was the last type of field pattern to be set up, as a result of the colonization of primarily mountainous regions. Like segmental plužina, type D offers a more recent adaptation to harsh environmental conditions. As the abundance of this type was strictly driven by the village layout, we assume that it was, in most cases, a result of planned colonization, without later land consolidation.

Field pattern *type E* (without internal division, and others) was characterized by a slightly lower altitude, while no other factors showed a significant effect. This type is present almost throughout the study area (with the exception of southern Bohemia, where there is a high occurrence of type B; Fig. 4E). Based on the preferred environmental conditions, we can describe this type as a “generalist”—it can tolerate a wide range of conditions without specific requirements. This is in accordance with the ecological observation that generalists usually do not need resource-rich habitats^187^. This type probably represents land owned by the nobility^24,182^.

Our findings show an evident link between the selected field pattern type and the settlement layout type (Table 3, Fig. 3). There is an apparent relationship between “villages with regular hides” and croft plužinas (notably, croft plužinas occur only in “villages with regular hides”). “Village green locations” are mostly connected with sectional plužina. “Towns” are primarily (although insignificantly) associated with plužina of (un)consolidated segments. “Dispersed” and “other” are not field-pattern-type specific. These findings partially correspond with previous research by Sadravetzová^88^: we have found an exact link between “villages with regular hides” and croft plužina. Unlike Sadravetzová, whose paper linked “village green locations” to segmental plužina, our results link “village green locations” to sectional plužina. This discrepancy may be caused by the different study areas (local *vs.* national). The linkage between settlement layout type and field pattern type was also supported by Dohnal and Škabrada^188^, who identified a clear relationship between the courtyard size and its hide width. Based on the sequence of houses and field parcels, similar results have also been reported from Northern Europe^91,189–191^.

Our data suggest a remarkable result (Table 2, Fig. 3): areas confiscated after the Thirty Years’ War have significantly different field pattern types (type C and maybe also type D) than areas that remained in the possession of their original landlords (type B). Types B and C occur in similar environmental conditions (high soil fertility, flat terrain). These two types could be different adaptations to the same requirements: rational, orthogonal organization of parcels. The difference between them lies in their spatial extent. While type B (plužina of (un)consolidated segments) requires only a limited area (e.g. 5% of the whole cadastre), type C (sectional plužina) needs at least one half of the cadastre area. We propose the following explanation of the observed data: As the confiscated cadasters were sold to new (foreign and wealthy) nobility, the new landlords probably had much more financial resources, foreign experience and willingness to reorganize the field pattern than the original owners [example: activities of Count von Sporck, son of the emperor’s general and a member of the “new” nobility, on his estates in Central and Eastern Bohemia^192,193^]. The new nobility was also catholic (as was the emperor), and the (primarily protestant) peasants may have been afraid to revolt in the times when the counter-reformation was beginning. These results also indicate that land consolidation activities also took place in the modern period, probably as a result of various land reforms^141^.

Contrary to the expectations suggested by Žemlička^83^, the variable *ancient settlement area* showed no significant effect. In other words, the presence of continuous settlement activities since the Neolithic played no role. This finding may seem rather surprising, as it has been considered that ancient settlement area had shared characteristics^129^.

Similarly, distance to *monasteries* was also an insignificant variable. Nevertheless, we cannot completely exclude the possible influence of medieval monasteries, which had some of their estates in distant parts of the country^194^.

Interestingly, several conditions are characteristic of one type of field pattern only. E.g. “villages with regular hides” are (in most cases) surrounded by croft plužinas only; extremely high terrain undulation is a precise predictor for segmental plužina; huge cadastres indicate the occurrence of sectional plužina, etc. Likewise, several regions are characterized by only one type of field pattern (Fig. 4). This finding is similar to observations from France, where typical regional forms of terraced landscapes have been identified^61^. Maybe the field pattern type was part of the specific regional traditions (like the vernacular architecture and the traditional folk costumes—cf.^195^)?

Our findings on the environmental predictors of type A (segmental plužina) are in contradiction to findings presented by Klír^24^ in his local study from western Bohemia. According to his results, the segmental plužina prefers the optimal conditions for agriculture. We think this discrepancy might be caused by local history or specific conditions in the region.

We should mention here several possible limitations of our study. First, the settlement selection was not random, but was based on the availability of archaeological data (see Fig. S1). This could theoretically pose a bias towards selecting specific geographical areas, and could thus affect the final results. Second, the field pattern types were determined by human researchers, and humans can make mistakes. Although we checked our data properly, we cannot exclude the possibility that the type of field pattern may have been categorized incorrectly in some cases. However, this should not play a role due to the relatively big sample. Third, the spatial extent of our study was limited to the western part of the Czech Republic by the availability of archaeological data. The generalization of our results is therefore disputable. It would open quite new possibilities to compare our results with findings from other countries, which is nevertheless dependent on the availability of standardized datasets.

## Conclusion

Our findings confirm the influence of environmental conditions, mostly terrain undulation and soil fertility, and the effect of geographical/cultural variables (e.g. cadastre size) on the field pattern type prevailing in a specific location. Moreover, we can suggest various “ecological adaptations” to specific environmental conditions (cf.^196^): Sectional plužina and plužina of (un)consolidated segments prefer good conditions for agriculture. Segmental and croft plužinas can withstand even very harsh conditions, and type E (others) is not linked to the conditions important for agriculture. These adaptations differ in the spatial extent of the field pattern type within a cadastre (sectional plužina *vs.* plužina of (un)consolidated segments); or age of origin, regional traditions and customary settlement layout type (segmental plužina *vs.* croft plužina) may have also contributed to the observed distribution. As suggested by previous studies, there is a clear link between field pattern type and settlement layout type. However, the link is valid for limited cases only (“village with regular hides”—croft plužina, “village green locations”—sectional plužina). This corresponds with ethnographic findings^91,188^. On the other hand, some older hypotheses (e.g. the influence of an ancient settlement area, proposed by Žemlička^83^) were not supported by our data.

Besides affecting the size of the village by providing an area for agriculture^137^, the size of the cadastre is also responsible for the number of field pattern types within the cadastre—and is therefore linked with cultural landscape diversity. The settlement density (i.e. the distance between villages) thus affected the village size and the overall landscape structure.

The type of field pattern has also been affected by seventeenth century confiscations: cadasters in confiscated estates have significantly different field pattern types than cadasters which remained in the possession of the original landlords. This phenomenon may have been caused by the non-local economic knowledge and the access to resources of the new nobility, allowing for re-organization of older field systems in accordance to the preferences of the impending landowners.

The spatial distribution of field pattern types within the country was probably also driven by other factors that were not included in our study. Strong regional prevalences of specific field pattern types can be observed (Fig. 4), which cannot be explained by environmental or geographical variables only. Theoretically, we could hypothesize that the field pattern type was a part of specific local/regional traditions (cf.^61^)—and that the adaptation to similar environmental (and other) requirements could differ from region to region.

Our findings do not dispute the results of previous historical research. Instead, we have confirmed earlier findings and have put them into a new, broader context. Together with historians and archaeologists, we can summarize that: During the early medieval period, the earliest plužina types were probably the segmental types (type A). Some plužinas were re-designed to sectional types (type C) during the high-medieval colonization (especially in areas with good conditions for agriculture). During the whole studied period (i.e. in medieval and early modern times), plužinas of consolidated/unconsolidated segments (type B) were popular as a rational way to consolidate the land-ownership structure. Croft plužinas (type D) were used during the late medieval colonization of sub-mountainous areas.

Apart from the environmental factors, our study shows a significant influence of historical/social factors (cadastre size, seventeenth century confiscations, settlement layout type, regional clustering). These predictors point to various historical turbulences through the centuries and their impact on the cultural landscape at the detailed scale.

To the best of our knowledge, our paper provides the first detailed analysis of the geographical distribution of traditional field systems at the scale of an entire state (the historical Kingdom of Bohemia). In addition to the environmental predictors, we want to stress the importance of social factors—e.g. the relationship with the settlement layout type, with the cadastre size, with the war disturbances and with the subsequent social unrest in the seventeenth century. It is crucial to study both types of driving forces and to take into account the mutual impacts of environmental and social factors.

## Supplementary Information

Supplementary Information 1.

Supplementary Figures.

## Data Availability

All used data are available in Dataset S1.
