# Author Correction: Ecological and historical factors behind the spatial structure of the historical field patterns in the Czech Republic

**DOI:** 10.1038/s41598-022-16238-8

**Published:** 2022-07-11

**Authors:** Václav Fanta, Jaromír Beneš, Jan Zouhar, Volha Rakava, Ivana Šitnerová, Kristina Janečková Molnárová, Ladislav Šmejda, Petr Sklenicka

**Affiliations:** 1 Faculty of Environmental Sciences, Czech University of Life Sciences Prague, Kamýcká 129, 165 00 Prague – Suchdol, Czech Republic; 2 Institute of Archaeology, Faculty of Philosophy, University of South Bohemia, Branišovská 31, 370 05 České Budějovice, Czech Republic; 3 Laboratory of Archaeobotany and Palaeoecology, Faculty of Science, University of South Bohemia, Na Zlaté stoce 3, 370 05 České Budějovice, Czech Republic; 4 Prague University of Economics and Business, W. Churchill Sq. 1938/4, 130 67 Prague 3 – Žižkov, Czech Republic; 5 Department of Anthropology, Faculty of Arts, University of West Bohemia, Univerzitní 8, 306 14 Plzeň, Czech Republic

Correction to: *Scientific Reports* 10.1038/s41598-022-12612-8, published online 23 May 2022

The original version of this Article contained an error in Reference 82,

82. Land Survey Office. Archive [web application]. *Central Archive of Surveying and Cadastre* https://ags.cuzk.cz/archiv/#en (2020).

now reads:

82. Imperial Imprints of the Stable Cadastre. *Central archive of surveying and cadastre*. Archive [web application].

https://ags.cuzk.cz/archiv#en (2020). *Aerial survey image, 1952—Archive of Aerial Survey Images of the Military Geographical and Hydrometeorological Office, Ministry of Defense of the Czech Republic,* Archive [web application]. https://ags.cuzk.cz/archiv#en (2020). *Orthophoto CR, 2018/2019/2021* Land Survey Office, Archive [web application]. https://ags.cuzk.cz/archiv#en (2020)."

The original Article has been corrected.

