# Supplementary material for: Ecological and historical factors behind the spatial structure of the historical field patterns in the Czech Republic
Source: Sci Rep. 2022 May 23;12:8645. doi: 10.1038/s41598-022-12612-8 (PMC9126947; doi:10.1038/s41598-022-12612-8)
Supplement: Supplementary file 2 — Supplementary Figures. [file 41598_2022_12612_MOESM2_ESM.pdf]

Supplementary information

for paper:

Fanta et al.: Ecological and historical factors behind the spatial structure of the historical field patterns in the Czech Republic

corresponding author:

Václav Fanta, fanta.vaclav@gmail.com, Faculty of Environmental Sciences, Czech University of Life Sciences Prague, Kamýcká 129, Praha – Suchdol, 165 00, Czech Republic

content:

Supplementary figure S1

Supplementary figure S2

Supplementary figure S1:

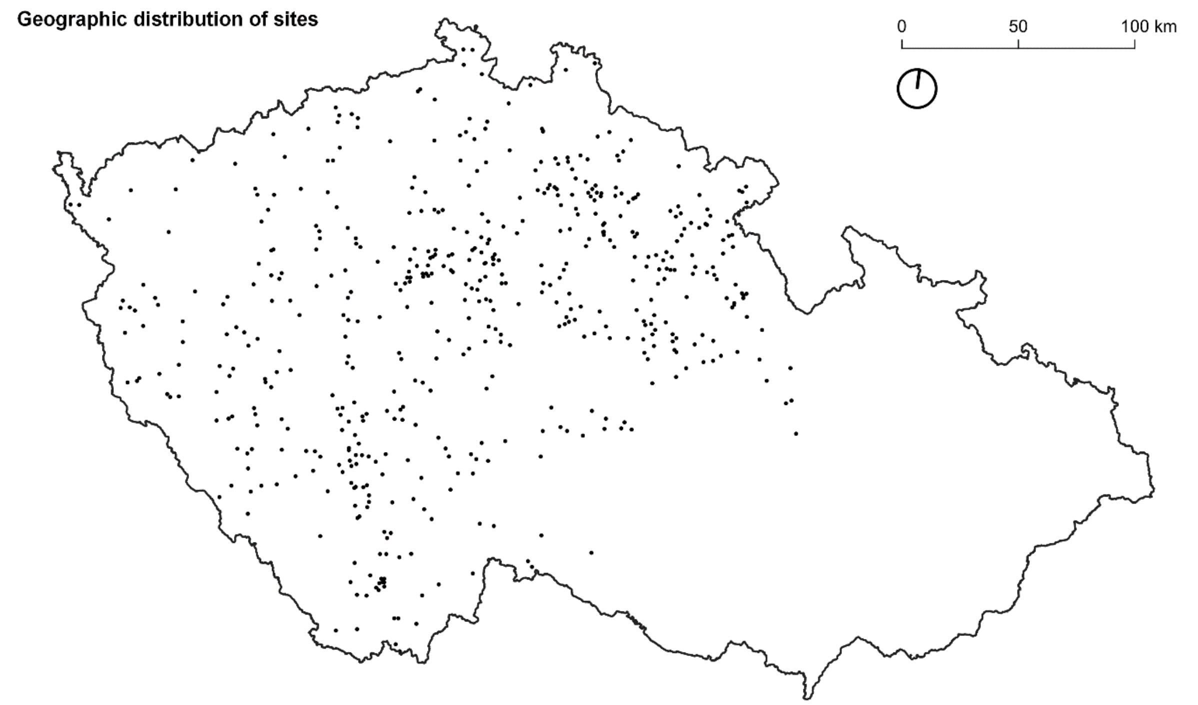

Fig. S1. Geographic distribution of examined sites within the Czech Republic. The map was created by authors using QGIS software (https://qgis.org/en/site/, version 3.22.0)

Supplementary figure S2:

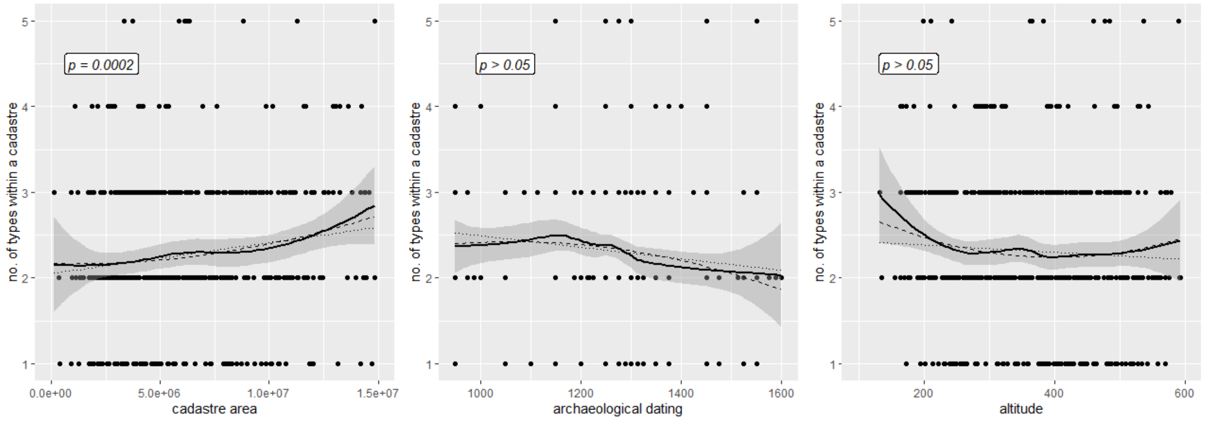

Fig. S2. Influence of *cadastre size*, *archaeological date* and *altitude* on the *diversity* of field pattern types
